# Two attentive strategies reducing subjective distortions in serial duration perception

**DOI:** 10.1371/journal.pone.0265415

**Published:** 2022-03-17

**Authors:** Franklenin Sierra, David Poeppel, Alessandro Tavano

**Affiliations:** 1 Department of Neuroscience, Max Planck Institute for Empirical Aesthetics, Frankfurt am Main, Hessen, Germany; 2 Department of Psychology, New York University, New York City, New York, United States of America; University of New England, AUSTRALIA

## Abstract

Humans tend to perceptually distort (dilate/shrink) the duration of brief stimuli presented in a sequence when discriminating the duration of a second stimulus (Comparison) from the duration of a first stimulus (Standard). This type of distortion, termed “Time order error” (TOE), is an important window into the determinants of subjective perception. We hypothesized that stimulus durations would be optimally processed, suppressing subjective distortions in serial perception, if the events to be compared fell within the boundaries of rhythmic attentive sampling (4–8 Hz, theta band). We used a two-interval forced choice (2IFC) experimental design, and in three separate experiments tested different Standard durations: 120-ms, corresponding to an 8.33 Hz rhythmic attentive window; 160 ms, corresponding to a 6.25 Hz window; and 200 ms, for a 5 Hz window. We found that TOE, as measured by the Constant Error metric, is sizeable for a 120-ms Standard, is reduced for a 160-ms Standard, and statistically disappears for 200-ms Standard events, confirming our hypothesis. For 120- and 160-ms Standard events, to reduce TOEs it was necessary to increase the interval between the Standard and the Comparison event from sub-second (400, 800 ms) to supra-second (1600, 2000 ms) lags, suggesting that the orienting of attention in time waiting for the Comparison event to onset may work as a back-up strategy to optimize its encoding. Our results highlight the flexible use of two different attentive strategies to optimize subjective time perception.

## Introduction

The processing of temporal information on the scale of milliseconds is essential to successfully navigate our sensory environment. Serial duration perception is a critical ingredient of complex motor and sensory phenomena such as speech recognition and motion processing [1–3]. One core capacity is the ability to compare and discriminate the duration of events in a series [4]. To test this ability in a simple, parametric, and quantitative manner, a two-interval forced choice (2IFC) paradigm can be employed in which a first event (the Standard event, S) is followed by a second event (the Comparison event, C), separated by an Inter-Stimulus Interval (ISI). The Comparison stimulus is generated by systematically adding or subtracting small fractions *Δt* to the Standard duration.

In a 2IFC test, individual temporal performance by sensory precision and perceived event duration, indexed via the Weber fraction (WF) and the Constant error (CE), respectively [5, 6]. The WF is a dimensionless measure of temporal resolution afforded by the sensory system, and is calculated as the ratio of the just noticeable difference (JND) to the duration of the Standard stimulus. The CE estimates the difference between the Point of Subjective Equality (PSE) and the duration of the S stimulus.

Distortions of perceived event duration are indexed by the signed magnitude of the CE. They are inherent to serial comparisons, and part of a more general, ubiquitous phenomenon termed Time Order Error (TOE, Fechner, 1860) [7]. Fechner first observed that in comparing the weight of two elements, a Standard and a Comparison, the order in which they were lifted mattered. The TOE was defined as positive if the first stimulus was overestimated, and negative if the second stimulus was overestimated. Vierordt’s (1868) work using an immediate interval reproduction task suggests that short intervals are overestimated, and long intervals are underestimated (as cited by Eisler, Eisler and Hellström [8]). Vierordt termed *Indifference intervals or Indifference points (IPs)* the durations which were veridically reproduced. When participants discriminate which of two successive events is longer (or shorter), they tend to dilate/shrink either or both events. The Differential Sensation Weighting model proposed by Hellström [9, 10] highlights the importance of the ISI factor in determining the type of subjective distortion: 1) short ISIs coupled with short event durations would lead to an “attentional blink” effect for both S and C, which however would preserve a larger sensory weight for the first stimulus (“primacy effect”); 2) longer ISIs–because of information loss due to increased memory demands—would lead to a reduced weight for the first stimulus relative to the second (“recency effect”). However, the available psychophysical evidence on the effects of ISI is mixed.

Buonomano and colleagues tested short (250 ms) and long (750 to 1000 ms) ISIs and showed that for a 100-ms auditory Standard temporal precision increases with long ISIs, while TOEs remained unaffected [11]. Insightfully, using both multiple and single Standard protocols, Grondin reported that the magnitude of the CE is markedly reduced if an ISI of about 1.5 seconds is used between Standard and Comparison [12]. This suggests that longer ISIs may benefit rather than hinder perception. From a temporal orienting of attention viewpoint, long ISIs should reduce time distortions since increasing the deployment of attention in time generally improves discrimination accuracy [13], likely improving the encoding of the Comparison event. Indeed, beneficial effects of the temporal orienting of attention—also termed foreperiod or waiting-time effects—on detection accuracy and response times have been documented for other tasks/sensory modalities [14, 15]. However, Bausenhart, Rolke & Ulrich [16] showed that a preparation time of 800 ms leads to better accuracy in a visual temporal order judgment (TOJ) task than a 2400-ms lag. To finely map how variations in the temporal orienting of attention modulate subjective time distortions, we parametrically varied the ISI between Standard and Comparison from sub-second to supra-second intervals: 400, 800, 1600, and 2000 m.

Grondin’s work is also suggestive of the existence of a veridical sweet spot for short visual stimulus durations: the Constant Error was positive and significant for a 150-ms Standard but tended to be suppressed or inverted in sign for a 300-ms Standard [12]. This qualitative change suggests the possibility of an optimal interval duration between 150 and 300 ms which, *per se*, would be sufficient to minimize time distortions independently of the ISI factor. The existence of a temporal sweet spot is predicted by rhythmic models of attention informed by neuroscience [17–19]. The central tenet of the rhythmic attention hypothesis is that intrinsic neuronal oscillations facilitate the chuncking and thus the processing of task-relevant information according to the internal oscillator’s phase [18, 20–23], avoiding sensory overflow and interference. In particular, Fiebelkorn and Kastner [20] proposed that rhythmic attention uses theta band activity (4–8 Hz) to temporally organize sensory and motor functions in the neural attention network. In order to behaviorally test such hypothesis, we mapped visual interval timing onto the theta band range by parametrizing the duration of the Standard event from 120 to 160, and then 200 ms, which would correspond to attentive frequencies of 8.33, 6.25, and 5 Hz, respectively. The 120-ms duration lies just outside the theta band, the 160-ms duration occupies its high portion, while the 200-ms lies in its core region. If fidelity in sensory processing depends on how well the incoming stimulus duration matches a participant’s endogenous attentive rhythm, centered on the theta band of the oscillatory spectrum, then group-wise behavioral temporal performance should be at ceiling for durations well within the theta band, regardless of any additional benefit that may come from the temporal orienting of attention (ISI factor). Conversely, the temporal orienting attention would be of the essence when moving outside of said sweet spot. Thus, we propose the existence of a dynamic trade-off between two attention strategies in duration perception.

## Materials and methods

### Ethics statement

The studies were approved by the Ethics Committee of the Max Planck Society. Written informed consent was obtained from all participants before each session.

### Participants

The experiment was organized as a between-subject design, with separate groups for each Standard (S) duration experiment. For the 120-ms S stimulus, the initial sample consisted of fifty-two participants (34 female; ages: 18–33; mean age: 24.42). For each participant we computed the goodness-of-fit of the psychometric function (*R*^2^). Participants with a *R*^2^ value lower than two standard deviations of the mean were removed. Six participants were removed from analysis following this procedure. Furthermore, we identified outliers for both dependent variables, WF and CE, by establishing an upper (UT) and a lower threshold (LT): UT = Q1–1.5 • IQR; and LT = Q3 + 1.5 • IQR, where IQR is the Interquartile Range, Q1 and Q3 the first and third quartile respectively. Seven participants were discarded following this additional procedure. Therefore, the final analysis for the 120-ms S stimulus included the data from thirty-nine participants (24 female; ages: 18–33; mean: 24.07). For the 160-ms S stimulus, we had an initial sample of fifty-three participants (42 female; ages: 18–34; mean age: 24.71). Five participants were removed from analysis due to their low *R*^2^ values. Eight participants were additionally discarded as WF/CE outliers. Therefore, the final analysis for the 160-ms S stimulus included the data from forty participants (31 female; ages: 19–34; mean: 25.47). For the 200-ms S stimulus, we had an initial sample of fifty participants (40 female; ages: 18–35; mean age: 24.54). Two participants were removed from analysis due to their low *R*^2^ values. Nine participants were discarded for being marked as WF/CE outliers. Therefore, the final analysis for the 200-ms S stimulus included the data from thirty-nine participants (32 female; ages: 18–31; mean: 23.94). In total, we report on the behavior of 118 participants.

Individuals were recruited through online advertisements. Participants self-reported normal or corrected vision and had no history of neurological disorders. Up to three participants were tested simultaneously at computer workstations with identical configurations. They received 10 euros per hour as a compensation for their participation.

### Design

**Fig 1:**
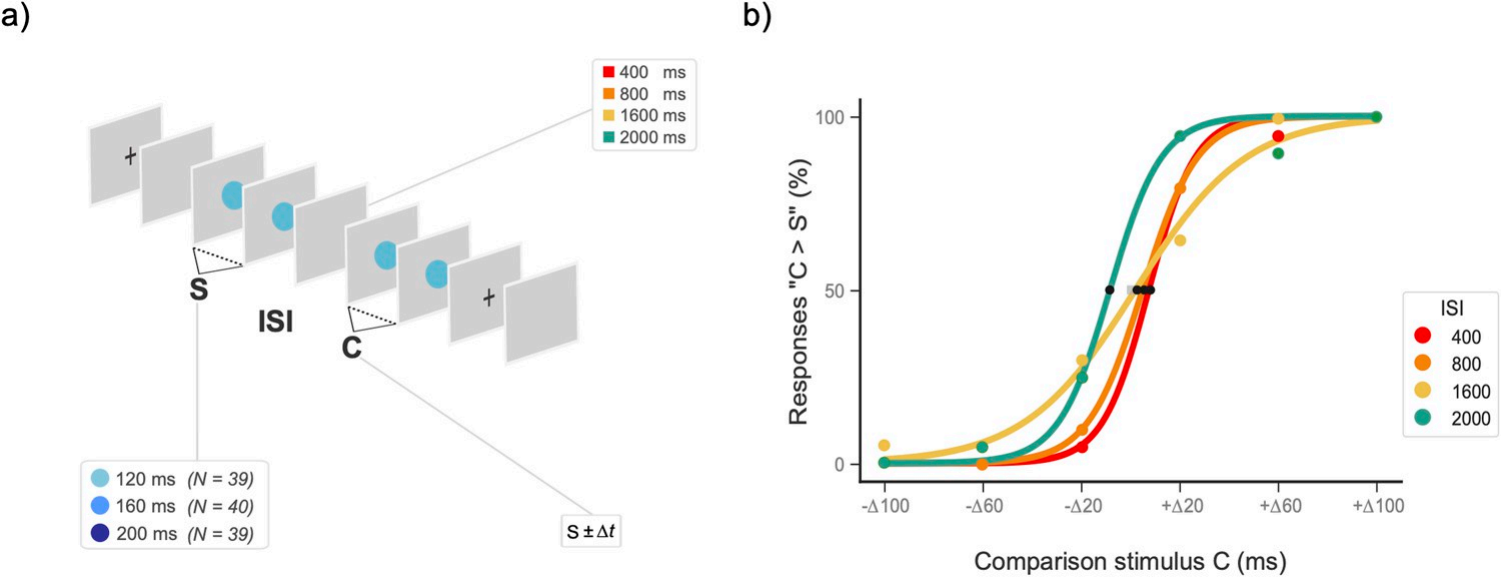
Experimental paradigm and response model. **a)** Sequence of events in the 2IFC temporal-discrimination task. To test whether rhythmic attention plays a role in time distortions, we parametrized the duration of S into three separate participant groups: 120-ms S, 160-ms S and 200-ms S, which correspond to attentive frequencies of 8.33, 6.25 and 5 Hz, respectively. For each S duration, the inter-stimulus interval (ISI) was parametrized into four levels: 400, 800, 1600, and 2000 ms. In each trial, participants determined whether the standard (S) or the comparison event (C) lasted longer. **b)** Example of response model fit for one participant in each ISI level of a 120-ms S: The six C durations are plotted on the x-axis and the probability of responding “C longer than S” on the y-axis. Black dots interpolated at the 50% value on the y-axis correspond to the points of subjective equality (PSE) for each condition. The just noticeable difference (JND) is obtained from the slope *β* of each curve.

We used a two-interval forced choice (2IFC) experimental design in a classical interval discrimination task [2]. For each S duration, participants were presented with a Standard (S) visual duration followed by a Comparison (C) visual duration, each defined by two successive flashes on each trial (Fig 1A). Participants judged whether S or C was the longer stimulus and responded by pressing one of two buttons on an RB-740 Cedrus Response Pad (*cedrus*.*com*, response time jitter ~ 1 ms, measured with an oscilloscope). They were provided with immediate feedback on each trial. Responses were modeled using a psychometric function (see Data analysis section; Fig 1B).

S always appeared in the first position (after the onset of a fixation cross followed by a blank interval; see Protocol), with a duration of 120 ms, 160 ms or 200 ms. For each S, we used three magnitudes for duration difference (*Δt*) between S and C: 20 (small), 60 (medium), and 100 ms (large). The durations of the C stimuli were derived as *S*±*Δt* (see Table 1).

**Table 1:** Standard (S) and Comparison (C) durations.

S duration (ms)	C durations (ms)					
120	20	60	100	140	180	220
160	60	100	140	180	220	260
200	100	140	180	220	260	300

Durations for the 2IFC temporal discrimination task. We used 3 levels for the S stimulus (between-subjects factor). The durations of the C stimuli were derived as *S*±*Δt*.

C duration was randomized on a trial-by-trial base. The inter-trial interval (ITI) was randomly chosen from a uniform distribution between 1 and 3 seconds. The same four different ISIs (400, 800, 1600, and 2000 ms) were used with each different S duration. The ISI intervals mapped onto both the sub-second and supra-second scales, thereby covering the range of potentially interesting intervals as per the literature [11, 12], and the assumption was a longer ISI would monotonically translate into TOE suppression. For each S duration, trial delivery was blocked by ISI.

### Stimuli and apparatus

Stimulus duration was determined as a succession of two blue disks with a diameter of 1.5^o^ presented on a gray screen. Empty stimuli were used to ensure that participants were focused on the temporal properties of the stimuli [24]. All stimuli were created in MATLAB R2018b (mathworks.com), using the Psychophysics Toolbox extension [25, 26]. Visual stimuli were displayed on an ASUS monitor (model: VG248QE; resolution: 1,920 x 1,080; refresh rate: 144 Hz; size: 24 in) at a viewing distance of 60 cm.

### Protocol (task)

Each participant completed a single session of about 70 minutes. Participants completed a practice set of four blocks (18 trials in each block). All sessions consisted in the presentation of four blocks, one for each ISI duration. Each block was composed of 120 trials; C durations were presented in random order for each ISI, and ISI blocks were also presented in a random orer. Each randomization was unique. To avoid fatigue, participants always had a break after 60 trials. Each trial began with a black fixation cross (diameter: 0.1^o^) displayed in the center of a gray screen. Its duration was randomly selected from a distribution between 400 and 800 ms. After a blank interval of 500 ms, S was displayed and followed by C after one of the ISI durations.

Participants were instructed to compare the durations of the two stimuli and press the key “left”, if S was perceived to have lasted longer, and the key “right” if C was perceived to have lasted longer. After responding, they were provided with feedback: the fixation cross color changed to green when the response was correct, and to red when the response was incorrect.

### Data analysis

The data analysis was implemented with Python 3.7 (python.org) using the libraries Pandas [27], Seaborn [28], Pingouin [29], and the ecosystem SciPy (scipy.org). Frequentist statistical analyses were executed in Pingouin. Bayesian statistical analyses were implemented using the BayesFactor package for R [30]. All data and statistical analyses were performed in Jupyter Lab (jupyter.org).

To support open science practices and transparency on statistical analyses [31], we used JASP (*jasp-stats*.*org*) for providing statistical results (data, plots, distributions, tables, and post hoc analyses) of both Frequentist and Bayesian analyses in a graphical user-friendly interface [32]. These results can be freely consulted at Open Science Framework as annotated.jasp files (*osf*.*io/583vg/*). As JASP uses the BayesFactor package [30] as a backend engine, the default prior distributions of the BayesFactor package were the same for JASP.

### Psychometric fit

A 6-point psychometric function was fitted to data of each participant, plotting the six Comparison durations on the *x* -axis and the probability of responding “C longer than S” on the *y* -axis. We modeled the psychometric function *ψ* with a logistic function *f*, with parameters *α* and *β*, so that:

$$\psi (x,\alpha ,\beta )=f(x)=\frac{1}{1\;+exp^{-(x-\alpha )/\beta }}$$

where *x* is the magnitude of the C stimulus, *α* is the location parameter, and *β* reflects the slope of the curve [33]. Fitting of the logistic function was done in Python using the nonlinear least-squares fit [34]. Two indexes of performance were extracted from each psychometric function: one for the perceived duration of the intervals, and another one for the discrimination sensitivity [35]. The perceived duration was measured based on the PSE, which is the point value on the *x* -axis corresponding to the 50% value on the *y* -axis (*α* in the logistic function). To normalize results, we obtained the CE as the difference between the PSE and the physical magnitude *ϕ*_*s*_ of the S stimulus (*CE* = *α*−*ϕ*_*s*_). An increase in the CE indicates that participants increased the duration of C for it to be perceptually equal to the S duration, an instance of time distortion or Time order error, TOE [36, 11]. In other words, positive CE values indicate that participants underestimate the C duration in comparison to the S duration.

We obtained the sensitivity for discriminating intervals by computing the JND, which is traditionally defined as being half the interquartile range of the fitted function: $JND=\frac{x_{.75}\;-\;x_{.25}}{2}$, where *x* ._75_ and *x* ._25_ denote the point values on the *y* -axis that output 25% and 75% “longer” responses [36, 37]. The smaller the JND, the higher the discrimination sensitivity of the observer. To compare the JND between S stimuli we obtained the WF as the ratio between the JND and the $\phi_{s}:\;WF=\frac{JND}{\phi_{s}}$ [38].

### Statistical analyses

For both dependent variables (i.e., the WF and the CE), we applied a mixed ANOVA using as factors S (between: three levels) and ISI (within: four levels). After that, in each S level we applied a repeated measures (RM) ANOVA across ISI levels (400, 800, 1600 and 2000 ms) to test for significant changes in both the WF and the CE. For the CE, we performed a series of Bonferroni-corrected ($\frac{\alpha }{4}$) one sample *t*-tests to zero to ascertain the presence of a significant effect at each ISI, within each S duration. We also applied linear and quadratic orthogonal polynomial contrasts to test the trend of the CE. The level of statistical significance to reject the null hypothesis was set to *α* = 0.05.

To characterize the strength of evidence in favor of the alternative model *M*_ISI_ (using the ISI factor as predictor) vs. the null model *M*_0_ (no difference among ISI levels), we implemented a Bayes factor approach to ANOVA by applying Bayesian Model Comparison [39–42]. To do that, we implemented Bayes’ rule [43, 44] for obtaining the posterior distribution *p*(*θ*|*Y*), where *Y* express the observed data. Under the model specification *M*_1_, the *p*(*θ*|*Y*) is given by

$$p(\theta |Y,M_{1})=\frac{p(Y|\theta ,\;M_{1})\;p(\theta |M_{1})}{p(Y|M_{1})}$$

where *p*(*Y*|*θ*, *M*_1_) denotes the likelihood, *p* = (*θ*|*M*_1_) express the prior distribution, and the marginal likelihood is expressed by *p*(*Y*|*M*_1_). In model comparison, we evaluate the predictive performance of two models. Thus, to evaluate the relative probability of the data under competing models, we estimated the Bayes factor (BF): Let BF_10_ express the Bayes factor between a null model *M*_0_ versus an alternative model *M*_1_. The predictive performance of these models is given by the probability ratio obtained by dividing the marginal likelihoods of both models:

$$BF_{10}=\frac{p(Y|M_{1})}{p(Y|M_{0})}$$

In this case, BF_10_ expresses the extent to which the data support the model *M*_1_ over *M*_0_, whereas BF_01_ indicates the Bayes factor in favor *M*_0_ over *M*_1_. BF values < 0 give support to *M*_0_, whereas BF > 1 support the *M*_1_ model [45]. A BF of 1 reveals that both models predicted the data equally well [46]. For our analysis of the dependent variables, we build an alternative model *M*_ISI_ that includes the ISI as predictor and compared it to the null model *M*_0_ (no difference among ISI levels). The prior model probability *p*(M) of each model was set to be equal, *i*.*e*., prior model odds of 0.5. For all our analyses we used the default prior values for Bayes factor ANOVA, which are also the default values in the BayesFactor package and JASP [41, 45].

## Results

### Goodness of fit (*R*^2^**)**

Participants were able to successfully discriminate between S and C durations in all experimental conditions: The goodness-of-fit of the psychometric function was high, making inferences on mental processes reliable. For the 120-ms S (8.33 Hz), the mean *R*^*2*^ values for the 400-ms, 800-ms, 1600-ms, and 2000-ms ISIs were 0.95, 0.97, 0.98, and 0.98 respectively. For the 160-ms S (6.25 Hz), the mean *R*^*2*^ values for were 0.98, 0.98, 0.98, and 0.98. For the 200-ms S (5 Hz), the mean *R*^*2*^ values were 0.97, 0.98, 0.98, and 0.98.

### Weber fraction (sensory precision)

We submitted the WF data to a Mixed ANOVA using as factors ISI (4 levels) as a within-subjects factor and Standard duration (3 levels) as a between-subjects factor. Results revealed significant effects of ISI and Standard duration (*F*(2.74, 315.32) = 11.11, *p* < 0.001, $\eta_{p}^{2}$ = 0.13, Huynh-Feldt correction; *F*(2, 115) = 15.32, *p* > 0.001, $\eta_{p}^{2}$ = 0.12; respectively). The interaction between the factors was significant (*F*(5.48, 315.32) = 3.59, *p* = 0.003, $\eta_{p}^{2}$ = 0.02, Huynh-Feldt correction). A mixed Bayesian ANOVA revealed that the data were best explained by the Model including both factors and their interaction (BF_10_ = 1.41 * 10^9^), when compared to the null Model.

**Fig 2:**
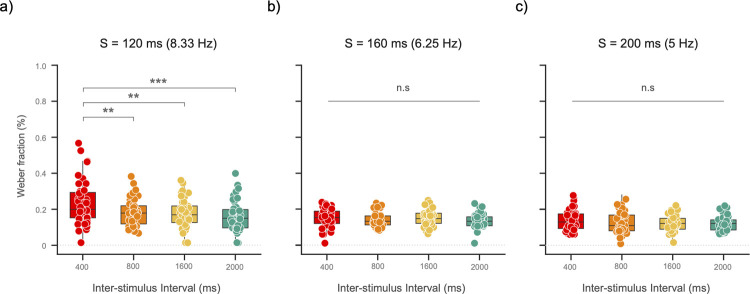
Sensory precision stabilizes at longer Standard events. Box-and-whisker plots show the distribution of the Weber fractions (WF) for each ISI level in each S duration. The bottom and top edges indicate the interquartile range (IQR), whereas the median is represented by the horizontal line. The extension of the whiskers is 1.5 times the IQR. Boxplots are overlaid with data of each participant. **a)** For a 120-ms S, results showed a significant effect of ISI: Temporal sensitivity increased for ISIs ≥ 800 ms, as reflected by lower WF values in the ISI_800_, ISI_1600_, and ISI_2000_ conditions. **b)** The effects of ISI disappeared for a 160-ms S. **c)** The same was true for a 200-ms S, as no changes between ISI levels were detected. (*** = *p*-value < 0.001; ** = a *p*-value < 0.01; and ‘n.s.’ = a non-significant result with a *p*-value ≥ 0.05).

We then analyzed ISI effects within each S duration. For a 120-ms S, the mean WF values for the 400-ms, 800-ms, 1600-ms, and 2000-ms ISIs were: 0.23 (SD = 0.12), 0.17 (SD = 0.07), 0.17 (SD = 0.08), and 0.16 (SD = 0.08). We found statistically significant differences among the four ISI levels (*F*(2.65, 101.03) = 8.98, *p* < 0.001, $\eta_{p}^{2}$ = 0.19, Huynh-Feldt correction). Post hoc comparisons showed that sensory precision in the ISI_400_ was lower than in the rest of the conditions: ISI_800_, ISI_1600,_ and ISI_2000_ (all *t*s ≥ 3.69, all *ps* ≤ 0.002, Bonferroni-corrected; Fig 2A). The Bayes Factor yielded decisive evidence in favor of the *M*_ISI_ model (BF_10_ = 935). That is, the data were 935 times more likely under the model that includes the ISI as a predictor, compared to the null model. (See annotated.jasp files with results of the Frequentist and Bayesian analysis at Open Science Framework: *osf*.*io/583vg/*).

For a 160-ms S, the mean WF values were: 0.15 (SD = 0.05), 0.13 (SD = 0.04), 0.14 (SD = 0.04), and 0.13 (SD = 0.04), for the 400-ms, 800-ms, 1600-ms, and 2000-ms ISIs conditions, respectively. The increase of 40 ms in S duration resulted in non-significant differences between ISI levels (*F*(3, 117) = 1.81, *p* = 0.148, $\eta_{p}^{2}$ = 0.04; Fig 2B). Congruently, the Bayes factor showed that the data were best explained by the *M*_0_ model (BF_10_ = 0.28).

For a 200-ms S, we again found no significant differences between ISI levels (*F*(3, 114) = 1.44, *p* = 0.235, $\eta_{p}^{2}$ = 0.03; Fig 2C). The mean WF values for the ISI levels were: ISI_400_ = 0.13 (SD = 0.05), ISI_800_ = 0.12 (SD = 0.06), ISI_1600_ = 0.12 (SD = 0.04), and ISI_2000_ = 0.12 (SD = 0.04). As for the 160-ms Standard, the Bayes factor showed that the data were best explained by the *M*_0_ model (BF_10_ = 0.18).

### Constant error (Time order error)

A mixed ANOVA for the CE revealed an effect of both factors (ISI and Standard duration), as well as their interaction (*F*(2.55, 293.94) = 24.61, *p* < 0.001, $\eta_{p}^{2}$ = 0.09; *F*(2, 115) = 3.35, *p* = 0.038, $\eta_{p}^{2}$ = 0.02; *F*(5.11, 293.94) = 3.34, *p* = 0.006, $\eta_{p}^{2}$ = 0.02; respectively; Huynh-Feldt correction). A mixed Bayesian ANOVA revealed that the data were best explained by the Model including both factors and their interaction (BF_10_ = 1.04 * 10^12^), when compared to the null Model.

**Fig 3:**
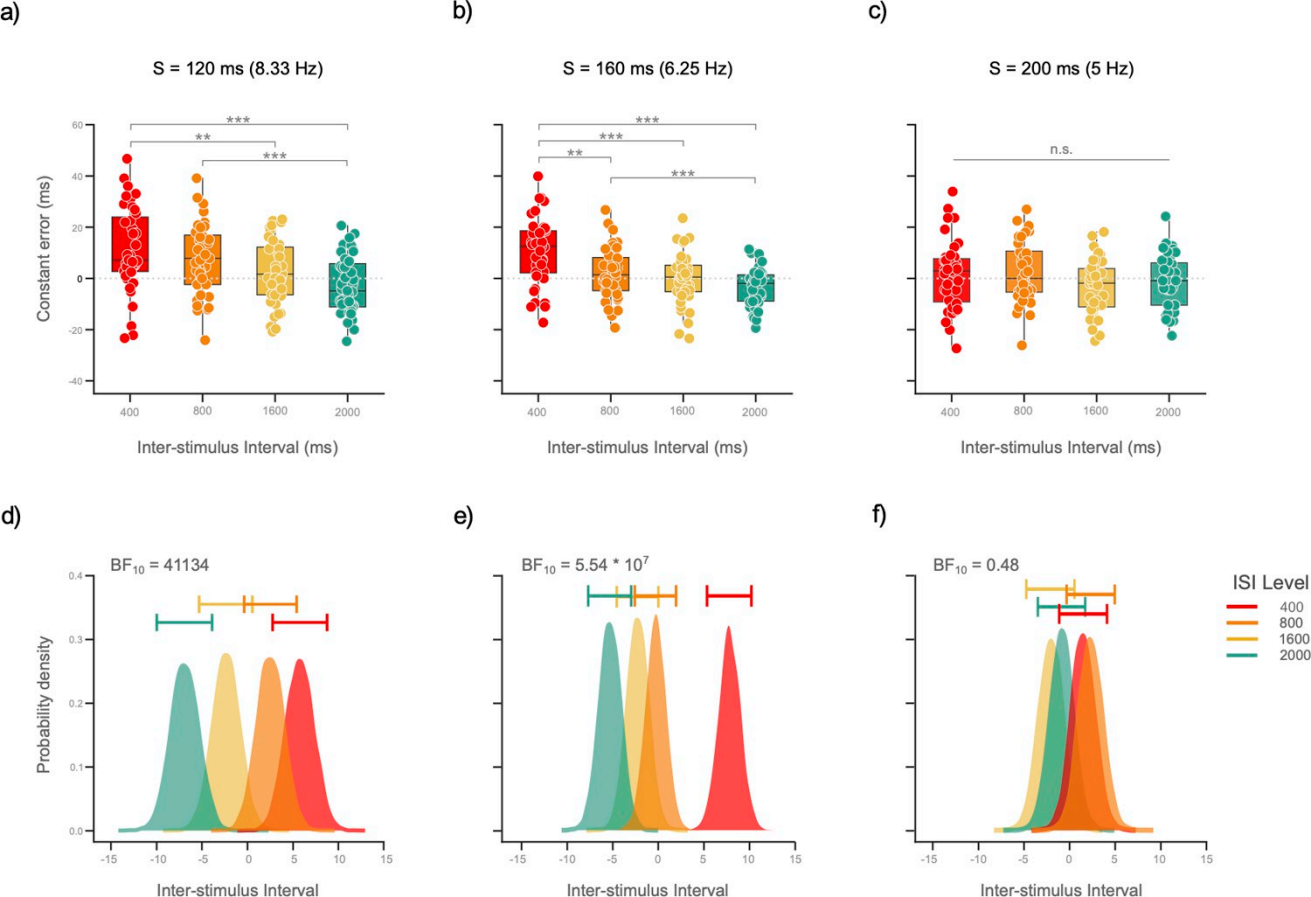
Time distortions are suppressed for longer Standard events. **a-c)** Box-and-whisker plots show the distribution of the Constant error (CE) for each ISI level in each S duration. **a)** With a 120-ms S (corresponding to an 8.33 Hz attentive rhythm) we observed a decrease of the CE as a function of ISI: ISIs longer than 800 ms decreased subjective distortions of the stimuli. **b)** The ISI effect was still present for a 160-ms Standard event (corresponding to a 6.25 Hz attentive rhythm), but this time limited to the ISI_400_ level. **c)** The effects of the ISI disappeared for a 200-ms Standard event (5 Hz attentive rhythm): No differences in CE among ISI levels were observed, and performance was group-wise not different from zero. In other words, for a 200-ms S event temporal perception was optimally achieved, independently of ISI. **D-f)** Results of the Bayesian ANOVA. Plots show the model-averaged posterior distributions (horizontal bars show the 95% credible intervals around the median). **d)** For a 120-ms S, posterior distributions showed a clear effect of the ISI. This effect is best expressed by the contrasting distributions of the two extreme levels: ISI_400_ vs ISI_2000_. **E)** Posterior distributions of a 160-ms S, showed again a clear separation between the ISI_400_ distribution and the rest of the conditions, which means that the beneficial effect of long ISIs is still present. **F)** Results of a 200-ms S, revealed no effect of ISI, as the overlap in posterior distributions visually confirms. (*** = *p*-value < 0.001; ** = a *p*-value < 0.01; and ‘n.s.’ = a non-significant result with a *p*-value ≥ 0.05).

For a 120-ms S, the mean CE values were: ISI_400_ = 11.21 (SD = 16.56), ISI_800_ = 7.63 (SD = 13.91), ISI_1600_ = 2.35 (SD = 12.52), and ISI_2000_ = -2.67 (SD = 11.37). We first tested the presence of TOEs by running a series of two-sided, one-sample *t*-test for each ISI level (Bonferroni corrected). We found a significant CE for both sub-second conditions, but not for the supra-second conditions (ISI_400_ and ISI_800_: *ts*(38) ≥ 3.42, *ps* ≤ 0.001; ISI_1600_ and ISI_2000_: *ts*(38) ≤ 1.17, *ps* ≥ 0.151; respectively). Congruently, the RM ANOVA showed a statistically significant difference between ISI levels (*F*(3, 114) = 12.44, *p* < 0.001, $\eta_{p}^{2}$ = 0.24), specifically between sub-second and supra-second conditions: ISI_400_ vs ISI_1600_; ISI_400_ vs ISI_2000_; and ISI_800_ vs ISI_2000_ (all *t*s ≥ 3.63, all *ps* ≤ 0.003; Bonferroni-corrected; Fig 3A). In line with these results, the Bayes factor revealed decisive evidence in favor of the *M*_ISI_ model (BF_10_ = 41134; Fig 3D).

For a 160-ms S, the mean CE values for the ISI levels were: ISI_400_ = 10.53 (SD = 13.12), ISI_800_ = 1.96(SD = 9.91), ISI_1600_ = -0.13 (SD = 10.03), and ISI_2000_ = -3.37 (SD = 7.33). One-sample t-tests showed that the ISI_400_ and ISI_2000_ conditions were significant, while the ISI_800_ and ISI_1600_ conditions were non-significant (*ts(*39) ≥ -2.9, *p* ≤ 0.006; *ts*(39) ≥ -0.085, *p* ≥ 0.217; respectively). We found again significant differences between ISI levels: *F* (2.21, 86.29) = 19.35, *p* < 0.001, $\eta_{p}^{2}$ = 0.33, Huynh-Feldt Correction. Post hoc analyses (Bonferroni correction) revealed that the ISI_400_ differed from all the remaining conditions (ISI_800_, ISI_1600_, and ISI_2000_), and that the ISI_800_ differed from the ISI_2000_ condition (all *t*s ≥ 3.55, all *ps* ≤ 0.006, Fig 3B). The Bayes factor reported decisive evidence in favor of the *M*_ISI_ model (BF_10_ = 5.54 * 10^7^; Fig 3E).

For a 200-ms S, the mean CE values for the ISI levels were: ISI_400_ = 1.28 (SD = 13.38), ISI_800_ = 2.30 (SD = 11.98), ISI_1600_ = -3.02 (SD = 10.48), and ISI_2000_ = -1.54 (SD = 10.35). One-sample t-tests found non-significant CE effects in all ISI conditions (all *ts*(38) ≥ -1.79, all *ps* ≥ 0.119). The RM ANOVA confirmed these results: *F*(2.64, 100.42) = 2.21, *p* = 0.099, $\eta_{p}^{2}$ = 0.05 (Fig 3C). Congruently, Bayesian analyses showed that the data were best explained by the *M*_0_ model (BF_10_ = 0.48; Fig 3F).

We implemented linear and quadratic polynomial contrast to further investigate the effects of S duration on the CE. Results revealed a significant linear trend (*t*(115) = -2.58, *p* = 0.011), whereas the quadratic fit was not significant (*t*(115) = -0.03, *p* = 0.972). This suggest that with increase in S, there is a linear decrease of the CE.

Taken together, our results suggest that setting the S stimulus to 200 ms minimizes TOEs in the context of high sensory precision, regardless of the ISI between S and C.

## Discussion

Interval timing is an essential feature of human behavior, and the errors one makes in discriminating the duration of two successive events depend on sensory precision and stimulus encoding fidelity. Sensory precision pertains to the resolution of the sensory system, in our case vision. Fidelity in stimulus encoding has been relatively less investigated, although it is known that humans tend to subjectively distort the perception of successive interval durations [7, 12]. To investigate how humans can minimize subjective distortions in duration perception, we parametrically manipulated two experimental features: The size of the inter-stimulus interval (ISI) between S and C as a proxy for the increase in temporal orienting of attention in time, [13, 14], and the size of Standard duration, embedding it within an assumed theta-band rhythmic attention sampling [18–20].

As for the ISI factor, Buonomano and colleagues observed that increasing ISI from 250 to 750 or 1000 ms with a 100-ms auditory Standard significantly improved sensory precision, measured as the percentage difference in stimulus duration that is just noticeable [11]. Although they used auditory rather than visual intervals, we expected in our results too an improvement in temporal sensitivity with longer ISIs. This prediction was borne out with a 120-ms Standard, but only when ISI was lengthened from 400 to 800 ms. As soon as the Standard was extended to 160 ms, sensory precision was at ceiling regardless of ISI. Hence, we conclude that sensory precision in duration discrimination is first and foremost a function of Standard duration. Notice that the absence of the temporal orienting of attention effect on sensory precision for 160-ms and 200-ms S is not, *per se*, a sufficient proof of optimal stimulus processing, as it could simply reflect a ceiling effect due to other factors, such as the use of empty intervals [24]. Rather, optimal processing is obtained when the stimuli are faithfully encoded regardless of ISI.

On this note, following the work of Grondin, an ISI of 1500 ms should lead to a reduction of TOEs [12]. However, Bausenhart et al. (2008) found that an ISI of 2400 ms is detrimental to temporal order judgement accuracy. We found that for 120-ms and 160-ms Standards, long ISIs (1600-ms and 2000-ms) benefited duration discrimination, minimizing subjective distortions in duration perception. We take this to suggest that increasing the orienting of attention in time with longer ISIs improves the encoding of the Comparison duration, with the caveat that such benefit may be reaching at upper bound at 2000-ms ISI.

With a 200-ms Standard corresponding to a 5-Hz attentive rhythm, optimal fidelity in stimulus encoding was obtained regardless of ISI, that is, according to our interpretation, independently of the temporal orienting of attention. We surmise that the 200-ms duration/5 Hz rhythm hits on a general sweet spot for rhythmic attentive processing, which would imply better time encoding fidelity for stimuli matching the attentive period. Alternatively, the 200-ms could reflect the emergence of a plateau in performance, with all stimuli equal to or longer than 200 ms being optimally processed: future work will adjudicate between these two stances. The 200-ms could be seen classically conceived of as an Indifference Point (IP), but because our stimuli were administered blocked by Standard duration, we cannot infer whether the 200-ms is a neurally hard-wired internal reference duration or is a perceptual bias that is updated on trial-by-trial basis [47].

Importantly, our results cannot be explained as a simple product of Weber’s law [48]. For 120-ms and 160-ms Standard stimuli, increasing temporal attention enhances temporal discrimination. Whereas for a 200-ms long Standard stimulus, temporal discrimination was at ceiling at each ISI level. According to Weber’s law [5, 48, 49], discriminating *Δ*20 for a 200-ms standard duration (that is, a 10% increase) should be more difficult—and consequently benefit more from temporal attention—than discriminating *Δ*20 for a 120-ms standard duration (a 16.6% increase). However, the opposite pattern occurred, strengthening the interpretation that a 200-ms standard event, corresponding to a 5-Hz rhythmic attentive rate, falls within an optimal time processing window.

The human brain dynamically may rely on different types of attentive functions depending on the characteristics of the stimulus to be encoded. Orienting attention in time captures an important but partial picture of how to go about obtaining sensory veridical percepts [13, 14]. Rather, sensory fidelity might depend on how well the incoming stimulus duration matches a participant’s endogenous attentive rhythm, centered on the theta band of the oscillatory spectrum. Electrophysiological results in animal research signal that the theta band would help segmenting sensory information in chunks amenable to processing [50–52]. Moreover, the theta rhythm appears to exert a general facilitatory function in internally representing complex events across perceptual domains, such as continuous speech [53, 54]. The underlying mechanism of rhythmic attention could be described as the alignment of a neural oscillation with stimulus onset, or a phase adjustment process, triggered in our case by phase reset due to flash onset. The visual system might be more prone than the auditory system to continuous adjustments to time-varying sensory input [19]. If a match between a hypothesized internal oscillator and external stimulus duration occurs, then even small differences in C will be encoded faithfully, leading to reduced TOEs. In this perspective, the effects of waiting time can be seen a backup mechanism, kicking in for small discrimination steps when stimulus durations are suboptimal for rhythmic attentive sampling functions. Naturally, while our novel behavioral results are in line with the existence of an oscillatory mechanism running at about 5 Hz and driving the attentive sampling of visual information, future research will have to test whether endogenous rhythmic attention indeed explains optimal processing for 200-ms stimuli. Another possibility would be to compare the results from a 2IFC with a time-reproduction task and verify if TOEs generalize to the motor domain [55], and if so whether the individual tendency to distort perceived durations is preserved across domains.

In sum, we provide evidence that visual stimulus duration is optimally processed at ~200 ms, with maximal fidelity in the subjective discrimination of serial durations, as well as in sensory precision. Orienting attention in time kicks in to help, should one stimulus duration be far from such optimal processing window.
